# Therapeutic nanoliposome vaccine targeting multiple Aβ and tau epitopes reduces AD-like brain pathologies and rescues cognitive deficits in 3xTg-AD mice

**DOI:** 10.1016/j.bbih.2025.101167

**Published:** 2025-12-30

**Authors:** Chun-Ling Dai, Yiting Song, Yonghua Chen, Yunn Chyn Tung, Wei-Chiao Huang, Cheng-Xin Gong, Jonathan F. Lovell

**Affiliations:** a Department of Neurochemistry, Inge Grundke-Iqbal Research Floor, New York State Institute for Basic Research in Developmental Disabilities, Staten Island, NY, 10314, USA; b Department of Biomedical Engineering, State University of New York at Buffalo, Buffalo, NY, 14260, USA; c POP Biotechnologies, Buffalo, NY, 14228, USA

**Keywords:** Active immunization, Vaccine, Aβ, Tau

## Abstract

Amyloid beta (Aβ) plaques and hyperphosphorylated tau neurofibrillary tangles (NFTs) are the histopathological hallmarks of Alzheimer's disease (AD) and targets for AD therapeutics. Since Aβ and tau pathologies both drive AD pathogenesis and progression, immunotherapies singularly targeting either Aβ or tau may be limited, and simultaneously targeting multiple epitopes of both Aβ and tau may be an efficacious approach. We developed a novel vaccine including three his-tagged tau peptides, tau_1-22_, mid-region tau_171-191_ (tau_pT181_), and tau_388-407_ (tau_pS396/S404_), as well as two his-tagged Aβ fragments (N-terminal Aβ_1-14_ and N-terminal pyroglutamate Aβ_pE3-14_) with the spontaneous nanoliposome antigen particle (SNAP) system, termed SNAP-AD_5_. Intramuscular vaccination of nine to ten months old of 3xTg-AD mice and age-matched wild-type control animals with SNAP-AD_5_ or adjuvant only, once every three weeks for a total of 5 immunizations, simultaneously produced IgG titers of antibody against their specific antigens, significantly decreased Aβ and tau pathologies, and effectively improved cognitive function. SNAP-AD_5_ was well tolerated without any detectable adverse side effects, including inflammatory responses in the peripheral circulation and in the brain, and hemorrhages in the mouse brain. These results support that SNAP-AD_5_ simultaneously targeting both Aβ and tau is potentially a promising new approach for treating AD. Further optimization and development of the SNAP-AD_5_ vaccine for treating AD is warranted.

## Highlights

• Simultaneously reducing amyloid beta (Aβ) and tau represents an efficacious approach.
• SNAP-AD_5_ vaccine produces antibodies targeting multiple epitopes of both Aβ and tau.
• Immunization with SNAP-AD_5_ decreases Aβ and tau and improves cognitive function.
• Immunization with SNAP-AD_5_ is well tolerated without any detectable side effects.
• SNAP-AD_5_ vaccine is potentially a promising new approach for treating AD.

## 1 Introduction

Alzheimer's disease (AD) is the most common cause of dementia and affects ∼6.9 million Americans over 65 years of age. The number of AD patients will increase to 13.8 million by 2060 if effective treatments are unavailable to prevent, delay, or treat the disease (Rajan et al., 2021). Extracellular senile plaques consisting of amyloid-β (Aβ) peptides (Glenner and Wong, 1984) and intracellular neurofibrillary tangles (NFTs) composed of abnormally hyperphosphorylated tau protein (Grundke-Iqbal et al., 1986) are the two major histopathological hallmarks in AD brains. Given their roles in the pathogenesis and progression of AD, preventing and/or removing Aβ and tau pathologies represents a promising approach for treating AD.

Active and passive immunotherapy targeting different forms of Aβ or tau are under active development (Dai et al., 2022). Because the central part of Aβ could induce T-cell response and cause severe side effects (Monsonego et al., 2003), the current Aβ immunotherapy targeting different N-terminus part of Aβ has shown beneficial effects in multiple mouse models and in the patients with early stage of AD. The US Food and Drug Administration (FDA) has approved the clinical use of aducanumab against Aβ_3-7_ in 2021, lecanemab against Aβ protofibrils_1–16_ in 2023 and donanemab against Aβ_pE3–7_ in 2024 for early AD. These approved monoclonal antibodies could effectively reduce Aβ plaques, but only slightly slow down the rate of cognitive decline in early AD patients and can not rescue the cognitive impairment. Biogen withdrew aducanumab from clinical use in 2024.

Immunotherapy targeting different epitopes and forms of tau has shown promising results in reduction of tau pathologies and improvement of cognitive function in mouse models, and the clinical trials are currently ongoing (Dai et al., 2022; Gallardo and Holtzman, 2017).

Given that Aβ and tau proteins synergistically drive the pathogenesis and progression of AD, the current immunotherapy approach targeting only one epitope of Aβ or tau may limit their clinical benefits. Therefore, developing a combinational therapy simultaneously targeting both Aβ and tau protein hold more promising for treating AD than current monotherapy. An active phase II/III clinical trial (ClinicalTrials.gov#NCT05269394) of combinational passive immunotherapy with lecanemab and E2814 against tau_273–291_ and tau_296–314_ in patients with early onset AD caused by genetic mutations is ongoing (Dai et al., 2022). Active immunotherapy with a dual Aβ/tau peptide vaccine against the N-terminus of Aβ and MTBR-tau could reduce Aβ plaque in APP/PS1 mouse (Barbour et al., 2024). Additionally, a MultiTEP vaccine containing a mixture of two vaccines: AV-1959R against Aβ_1-11_ and AV-1980R against tau_2-18_ reduced both Aβ and tau pathologies in Tau22/5xFAD mice (Davtyan et al., 2019). Vaccination with an Aβ peptide vaccine against Aβ_1-6_ and a phosphorylated tau peptide vaccine encompassing four phosphorylation sites (pS202/T205/S396/S404) exhibited beneficial effects in 3xTg-AD mice (Feng et al., 2024).

We previously developed an adjuvant and antigen delivery system, termed spontaneous nanoliposome antigen particle (SNAP) (Huang et al., 2018). The SNAP vaccine system works on the basis of cobalt porphyrin anchoring peptides with short his tags onto the surface of immunogenic liposomes (Shao et al., 2015a). We recently reported a SNAP vaccine containing a mosaic mixture of short his-tagged Aβ_1-14_, pyroglutamate-modified Aβ (Aβ_pE3-14_), tau_171-191_ (Tau_pT181_), tau_207-227_ (Tau_pT217_), and tau_388-407_ (Tau_pS396/S404_) peptides with liposomes containing CoPoP/PHAD, which demonstrated prophylactic activity in 3xTg-AD mice (Song et al., 2024). We found that the SNAP-AD vaccine produces high antibody titers against its specific antigens, reduces both Aβ and tau pathology and prevents cognitive decline without side effects during the 39-week study. The elicited mouse antibodies recognized Aβ plaques and NFTs in human AD brain sections (Song et al., 2024) has also demonstrated potential for human translation, with encouraging results observed in a COVID-19 vaccine that progressed through successful Phase 3 clinical trials (Lovell et al., 2022, 2024a, 2024b).

Since our group has found passive immunotherapy with tau antibody 43D against tau_6-18_ could reduce tau and Aβ pathologies, improve cognitive function, and block the propagation of tau pathology templated by AD P-tau in 3xTg-AD mice (Dai et al., 2017, 2018), we modified our SNAP-AD_5_ vaccine by adding a peptide of tau_1-22_ and removing the peptide of tau_207-227_ because of its relative low immunogenicity in our previous study (Song et al., 2024). Here, we studied the therapeutic efficacy of the new vaccine, referred to herein as SNAP-AD_5_, in 3xTg-AD mice by starting immunization at 9-10-months of age, followed by four boosts with a 3-week interval. SNAP-AD_5_ elicited high antibody titers against its specific antigens, significantly improved cognitive function and reduced Aβ and tau pathologies. Immunization with SNAP-AD_5_ vaccine was well tolerated and did not cause any detectable cerebral hemorrhages or inflammatory response in mouse brains or in the periphery.

## 2 Materials and methods

### 2.1 Reagents and antibodies

The his-tagged peptides Aβ_1-14_ [DAEFRHDSGYEVHH-HH], Aβ_pE3-14_ [*p*EFRHDSGYEVHH-HH, N-terminal pyroglutamate-modified], Tau_n1-22_ [HHHH-MAEPRQEFEVMEDHAGTYGLGD], tau_pT181_ [HHHH-IPAKTPPAPK(phospho-T)PPSSGEPPS], tau_pS396/S404_ [HGAEIVYK(phospho-S)PVVSGDT (phospho-S)PRH-HHH] peptides were synthesized by GenScript. CoPoP was prepared according to previously described methods (He et al., 2021a; Shao et al., 2015a). CoPoP liposomes were synthesized using the following lipids: CoPoP, 1,2-dipalmitoylsn-glycero-3-phosphocholine (DOPC, Corden # LP-R4-078), cholesterol (PhytoChol, Wilshire Technologies), Monophosphoryl Hexa-acyl Lipid A, and 3-Deacyl (Synthetic PHAD-3D6A, Avanti Cat # 699855P, abbreviated herein as PHAD). The anti-mouse HRP-linked IgG (CellSignaling, # 7076S), and 3,3′,5,5′- tetramethylbenzidine solution (Surmodics, TMBW-0100-01) were used for ELISA. Mouse IFN-γ Single-Color ELISpot kit (ImmunoSpot, Cat# mIFNgp-2M/10) and Mouse IgG Single-Color ELISpot kit (ImmunoSpot, Cat# mIgG-SCE-2M/10) were purchased for ELISpot assays. The primary antibodies 43D against transgenic human tau and R134d against total tau was generated in our laboratory. Primary antibodies including Tau_pT181_ (Invitrogen, Cat #701530), Tau_pS199_ (Invitrogen, Cat#44-734G)**,** AT8 (Invitrogen, Cat#MN1020), Tau_pT212_ (Invitrogen, Cat# 44-740G), Tau_pT217_ (Invitrogen, Cat #44–744), and Tau_pT231_ (Invitrogen, Cat #44-746G), and human Aβ 40 ELISA kit (Invitrogen, Cat#KHB3481) and human Aβ 42 ELISA Kit (Invitrogen, Cat #KHB3441) were obtained from ThermoFisher Scientific. PHF1 antibody is a kindly gift from Dr. P. Davies, Albert Einstein College of Medicine (Bronx, NY, USA). Anti-GAPDH antibody GAPDH antibody (Cat#G8795) and GFAP antibody (AB#5541) was obtained from MilliPoreSigma. Aβ antibody (D54D2) was obtained from Cell signaling (Cat#G8243). Iba 1 antibody was obtained from Abacam (Cat#AB289874).

### 2.2 Vaccine preparation

CoPoP/PHAD liposomes were made using ethanol injection, and followed by nitrogen-pressurized lipid extrusion through polycarbonate membrane filters in phosphate-buffered saline (PBS) as previously described (Shao et al., 2015b). The CoPoP/PHAD (CP) liposome formulation consisted of a lipid mass ratio of [DOPC:CHOL:PHAD:CoPoP] [20: 5: 0.4: 1], while PoP/PHAD liposomes was formulated with [DOPC:CHOL:PHAD:PoP] [20: 5: 0.4: 1]. Initially, lipids were dissolved in 55 °C ethanol for 10 min sonication, and then re-dissolved in 55 °C PBS for additional 10 min. Liposomes were extruded multiple times through stacked polycarbonate membrane filters (200, 100, and 80 nm) under nitrogen pressure using a lipid extruder [Northern Lipids]. Liposomes were further dialyzed twice in PBS at 4 °C to remove excess ethanol. Dynamic light scattering (DLS) with a NanoBrook 90Plus PALS instrument was used to measure liposome sizes and polydispersity index (PDI) of samples after 500-fold dilution in PBS. Zeta potential of liposomal antigen was measured in DI water with Malvern Zetasizer as previously described (He et al., 2021b). CoPoP or PoP/PHAD liposomes at 320 μg/mL and 5 peptides (Aβ_1-14_, Aβ_pE3-14_, tau_n1-22_, tau_pT181_, and tau_pS396/S404_) at 80 μg/mL were admixed and incubated at a 4:1 mass ratio for 3 h at 37 °C. For the immunization study, we prepared and diluted to the final concentration of 10 μg of multiplexed antigens (2 μg per individual antigen) combined with 40 μg of CoPoP liposomes with PBS in a 50 μL injection volume per mouse. All vaccines were further frozen at −20 °C, and shipped to New York State Institute for Basic Research.

### 2.3 Microfiltration binding assay

Both individual and multiplexed antigens of 2 μg dose were incubated with either CoPoP/PHAD or PoP/PHAD liposome at 37 °C for 3 h. The binding percentage was carried out with a micro-centrifugal filtration assay using bicinchoninic acid assay (BCA, Fisher catalog# PI23235). The incubated liposome-antigen mixture was diluted in PBS to a final volume of 200 μL, and centrifuged at 1200 g for 1 h at room temperature in a 100 kDa molecular weight cut-off centrifugal tube (PALL). The unbound peptides were collected in the supernatant, and further mixed with BCA reagent in a 1:1 vol ratio at 60 °C for 30 min. A 100 μL sample of this mixture was transferred in triplicate into a 96-well plate, and absorbance was recorded at 562 nm. The binding percentage was calculated using the following formula: Peptide Binding (%) = [1-OD562 of filtered liposome and peptides/OD562 of filtered peptides in supernatant only] × 100 %.

### 2.4 Immuno-slot blot assay

To investigate the structural integrity of SNAP-AD_5_ in particle form with liposome, we set up a 48-well slot apparatus (Bio-Rad, catalog# 1706542) with the provided instruction. Initially, 50 μL of PBS was applied to each well to check for leakage. Next, 100 μL of the diluted samples (10 ng/mL) were loaded onto a pre-wetted 0.2-μm nitrocellulose membrane (ThermoFisher, catalog# 77012). After blocking with 5 % BSA in PBS for 1 h at room temperature, the membrane was cut into individual strip, and applied with specific primary antibodies in 5 % BSA in PBS for 1 h. The antibodies are listed with following dilution ratio (1) anti-Aβ clone 6E10 at 1:2500 (Biolegend, catalog# 803004), (2) anti-Glu3 clone 337.48 at 1:2500 (Biolegend, catalog# 822301), (3) 43D at 1:2500 (provided by New York State Institute), anti-Tau Phospho (Thr181) clone M7004D06 at 1:2500 (Invitrogen, catalog# 710561), and anti-Tau Phospho (Ser396/Thr404) clone PHF-1 (provided by Albert Einstein College) at 1:5000. After wash with PBS three times, the strips were incubated with anti-mouse (Cell Signaling, catalog# 7076S) or anti-rabbit (Cell Signaling, catalog# 7074S) HRP-conjugated secondary antibodies (1:2000 in 5 % BSA) for 30 min. Following two washes in PBS, VisiGlo HRP substrate mixture (VWR, catalog# 97063–148) was applied to the membrane. Imaging was conducted using the Bio-Rad ChemiDoc™ Imager.

### 2.5 **E**LISA

The 96-well plates were coated with 2.5 μg/mL of Aβ_1-42_, Aβ_pE3-14_, tau_n1-22_, tau_pT181_, or tau_pS396/S404_ peptides in coating buffer containing 0.1 M carbonate/bicarbonate (pH 9.6) for 1 h at 37 °C. Plates were washed with PBST (0.05 % Tween-20 in PBS) and blocked with 2 % BSA in PBST for 1 h at 37 °C. Serial dilutions of 100 μL mouse serum were added and incubated for 1 h at 37 °C, followed by HRP-conjugated anti-mouse IgG (1:2000) for 30 min. After addition of TMB substrate, the reaction was stopped with 1 M HCl, and absorbance was recorded at 450 nm. IgG titers were defined as the reciprocal dilution giving an OD450 that exceeded background by 0.5 units. For IgG quantification, antigen-specific concentrations were determined using a standard curve generated with commercial monoclonal antibodies: anti-Aβ clone 6E10, anti-Glu3 clone 337.48, anti-Tau clone 43D, anti-phospho-Thr181 clone M7004D06, and anti-phospho-Ser396/Thr404 clone PHF-1. Concentrations were calculated relative to these standard curves.

### 2.6 T-cell IFN-γ enzyme-linked immunosorbent spot (ELIspot) assay

Mouse splenocytes cell were harvested and analyzed using a mouse IFN-γ single-color ELISpot followed with the manufacture's protocol. Mice were either not immunized or immunized intramuscularly with 50 μL of individual peptide or SNAP-AD5 on day 0, day 21, and euthanized for splenocyte's collection on day 42. Splenocytes were mashed through a 70 μm cell strainer, centrifuged at 500 g for 5 min, and treated with 5 mL red blood lysis buffer on ice for 5 min. Cells were then washed with PBS, centrifuged again, and resuspended in 1 mL PBS. For ELISpot, 3 × 10^5^ splenocytes were seeded per well and stimulated with 10 μg/mL of individual peptide, 5-plex, or media alone for negative control in a 96-well plate for 24 h at 37 °C in a 5 % CO_2_ incubator. Spots were imaged and counted using a CTL ImmunoSpot S6 FluoroCore analyzer.

### 2.7 B-cell IgG ELIspot

Mouse blood samples were collected through submandibular puncture and processed using a mouse IgG single-color ELISpot kit according to manufacturer's protocol. Mice were either not immunized or immunized intramuscularly with 50 μL of SNAP-AD5 on day 0, day 21, and collect blood on day 42. Blood was treated with 5 mL red blood lysis buffer for 5 min on ice and two washes with PBS by centrifugation. A total of 6 × 10^5^ cells were stimulated overnight with B-Poly-S™ and seeded into 96-well plates pre-coated with either anti-Igκ and anti-Igλ antibodies, 5-plex peptides or medium. The plate was incubated for 20–24 h at 37 °C in a humidified 5 % CO_2_ incubator. Spots were imaged and counted using a CTL ImmunoSpot S6 FluoroCore analyzer.

### 2.8 Reverse-phase HPLC (RP-HPLC)

RP-HPLC analysis on peptides was performed using a Waters Symmetry 300™ C18 analytical column (2.1 mm × 100 mm, 300 Å) maintained at 20 °C. Peptide samples were diluted to 150 μg/mL in DI water, and 30 μL of each sample was injected per run. Peptide separation was carried out at 1 mL/min flow rate with following gradient method: mobile phase A: 0.05 % Trifluoroacetic Acid (TFA) in distilled water, mobile phase B: 0.05 % Trifluoroacetic Acid (TFA) in 100 % Acetonitrile, 0 min–5 % B, 10 min–35 % B, 12 min–95 % B, 15 min–95 % B, 20 min–5 % B, 25 min–5 % B.

### 2.9 Immunization of mice

Female CD-1 mice (5–6 weeks old) were immunized intramuscularly with either 50 μL of SNAP-AD5 or 50 μL of 2 μg individual peptide vaccines on day 0 and 21, and serum (centrifuged at 2000 g for 20 min) were collected on day 42.

The homozygous 3xTg-AD mice (Strain#004807) and B6129SF2/J mice (Strain#101045) used as wild-type (WT) controls were originally obtained from the Jackson Laboratory (Oddo et al., 2003), and bred in pathogen-free facilities with 12-h light/12-h dark cycles in the animal colony of New York State Institute for Basic Research in Developmental Disabilities. The female 3 × Tg-AD mice develop amyloid plaques starting at about 9 months of age and NFTs starting about 12 months of age, respectively, and the pathologies are predominantly restricted to the hippocampus, amygdala, and cerebral cortex (Oddo et al., 2003). 3xTg-AD and WT mice (9–10 months old) were initiated the immunization followed by 4 boosts with a 3-week interval. The animals were intramuscularly injected with 50 μl of SNAP-AD_5_ vaccine containing 2 μg peptide of each antigen, or SNAP only without antigen as control per immunization. Male 3xTg-AD mice are not include in the present study because female 3xTg-AD mice exhibited consistent and overt AD-like pathologies and worse cognitive impairments than male 3xTg-AD mice (Barber et al., 2024; Carroll et al., 2010; Clinton et al., 2007; Wei et al., 2020). The general condition was monitored, and body weight was measured once a week during the period of the study.

### 2.10 Open field test

The open field test, used to assess locomotion, exploration and anxiety in mice (Chen et al., 2014; Walsh and Cummins, 1976), was conducted as previous report (Pan et al., 2021; Song et al., 2024). Briefly, each mouse freely explored the open field arena (50 cm × 50 cm × 40 cm) for 15 min. The distance travelled (meters) in open field arena, the entries into central area, time spent in the central area, and distance travelled (%) in the central area (10 cm × 10 cm), were automatically recorded by a video tracking system (Any maze version 4.5 software, Stoelting Co.)

### 2.11 One-trial novel object recognition task

One-trial novel object recognition test was performed as described previously (Dai et al., 2017; Pan et al., 2021). Briefly, the animal was allowed to explore the open field arena for 5 min in which two identical objects were placed after four consecutive days habituation. Then, the animal was returned to its home cage. Twenty minutes later, the animal was allowed to explore the open field arena for 5 min in which one of the familiar objects was replaced by a novel object. ANY-maze video tracking system (Version 4.5; Stoelting Co., Wood Dale, IL, USA) was used for recording and collecting the data. The object discrimination index was calculated as follows: [(time spent exploring the novel object)/(time spent exploring both familiar and novel objects) × 100 %] during the test phase.

### 2.12 Morris water maze task

Morris water maze (MWM) test used to evaluate spatial learning and memory of the mice was performed as described previously (Dai et al., 2017; Pan et al., 2021; Song et al., 2024). Briefly, each animal conducted 4 trials per day for 4 days to find the hidden platform in a pool with a 180 cm diameter. The animal was gently guided to the platform and stayed on the platform for 15 s if it failed to find the platform within 90 s training. Each animal conducted a 90 s probe test without platform on 5th day. Escape latency (sec) in initial training, and time spent in target quadrant (sec), latency to first entrance into target (platform location area), target crossings, and swimming speed (cm/sec) in probe test were recorded through Smart video tracking system (version 2.0.14, Panlab; Harvard Apparatus, Holliston, MA, USA).

### 2.13 Tissue processing

All mice were sacrificed by cervical dislocation. Forebrain cortex and hippocampus were detached immediately from the right hemisphere and froze in dry ice, and stored at −80 °C for biochemical analysis. The left hemisphere was fixed in 4 % paraformaldehyde in 100 mM phosphate buffered saline (PBS) for 24 h at 4 °C. Tissues were then post-fixed in a 30 % sucrose solution at 4 °C for overnight. Forty μm sagittal sections of the entire half hemisphere were cut using a freezing microtome. The sections were stored in glycol anti-freeze solution (ethylene glycol, glycerol and 100 mM PBS in 3:3:4 ratio) at −20 °C until further processing.

### 2.14 Mouse serum collection

Blood was collected via cardiac puncture immediately after cervical dislocation at the experimental endpoint and was storted at 4 °C for 1 h. The serum was collected by centrifuge at 1000 *g* × 10 min at 4 °C. The serum was aliquoted for antibodies titers and cytokines analysis (Creative Proteomics, NY, USA).

### 2.15 Cytokines and chemokines analysis in mouse serum

Preinflammatory and anti-inflammatory cytokines, and preinflammatory chemokines in mouse serum were quantified by Luminex multiplex assay by Creative Proteomics (New York, USA) as previous report (Song et al., 2024).

### 2.16 Western blot analysis

Mouse hippocampus was homogenized in pre-chilled buffer containing 50 mM Tris–HCl (pH 7.4), 100 mM NaF, and 1 mM Na_3_VO_4_, 1 mM EGTA, 0.5 mM AEBSF, 10 μg/ml aprotinin, 10 μg/ml leupeptin, 10 μg/ml pepstatin. Each homogenate was boiled in 2 × Laemmli's buffer for 10 min, and protein concentration was measured by Pierce™ 660 nm protein assay (Thermo Scientific, Rockford, IL, USA) (Song et al., 2024). The samples were separated by 10 % or 12 % SDS-PAGE and electro-transferred onto Immobilon-P membrane (Millipore, Bedford, MA, USA). The blots were then probed with primary antibodies and developed with the corresponding horseradish peroxidase–conjugated secondary antibody and ECL kit (Pierce, Rockford, IL). Densitometrical quantification of protein bands in Western blots were analyzed using identical background subtraction parameters applied uniformly to all lanes, rather than lane-specific adjustments by using the Multi Gauge V3.0 software (Fuji Photo Film Co., Ltd) as described in previous report (Song et al., 2024). Western blot and quantification were performed by an investigator blinded to experimental group identity.

Cortical sarkosyl-soluble and *-*insoluble tau were prepared by homogenization of cortical tissue with buffer containing 50 mM Tris-HCL, 2.0 mM EGTA, 2.0 mM Na3VO4, 50 mM Na3VO4, 0.5 mM AEBSF, 10 μg/ml Aprotinin, 10 μg/ml Leupeptin, 10 μg/ml Pepstatin. After incubation with 1 % N-lauroylsarcosine and incubated for 1 h at room temperature, the homogenates were centrifuged at 200,000 g for 45 min at 25 °C (Diner et al., 2017). The supernatant (sarkosyl soluble) and the pellet (sarkosyl insoluble) was dissolved in Laemmli sample buffer and subjected to Western blot analysis.

### 2.17 Immunofluorescence and thioflavin S staining

The Aβ plaques in the mouse brain was analyzed as previous report (Song et al., 2024). Briefly, serial 6–7 free-floating sagittal sections per mouse were washed in 10 mM PBS (3 × 15 min) and then incubated in 0.3 % Triton X-100 for 30 min. The sections were again washed in 10 mM PBS and blocked by incubation in blocking solution (5 % normal goat serum, 0.1 % Triton X-100, and 0.05 % Tween 20 in PBS) for 60 min. Sections were then incubated with AT8 (phospho-tau at Ser202/Thr205, 0.2 μg/ml; Thermo Fisher Scientific, Rockfork, IL, USA) or both PHF-1 (phospho-tau at Ser396/404, 1:1000, a gift from Dr. Peter Davies) and rabbit monoclonal antibody against Aβ (D54D2, 1:1000; Cell Signaling Technology, Danvers, MA, USA) at 4 °C overnight. On the second day, sections were washed in 10 mM PBS (3 × 15 min), followed by incubation with Alexa Fluor 555-conjugated goat anti-mouse IgG (1:500; Thermo Fisher Scientific), or in case of double immunostaining, in combination with Oregon Green 488-conjugated goat anti-rabbit IgG (1:500; Thermo Fisher Scientific), in 10 mM PBS with 0.05 % Tween 20 for 2 h at room temperature. Sections were subsequently incubated with DAPI (1 μg/ml) for 5 min, and washed, mounted onto Superfrost Plus slides (Fisher Scientific, Pittsburgh, PA, USA). The sections incubated with both PHF1 and Aβ antibodies were cover slipped with ProLong Gold Antifade Mounting Medium (Thermo Fisher Scientific). The sections incubated with AT8 antibody were dried overnight at room temperature. Sections were stained with 0.05 % thioflavin-S in water in dark for 8 min, and then were washed in 80 % ethanol for 2 min and in water for 3 min, and cover slipped using prolong gold antifade reagent from Thermo Fisher Scientific Inc (Waltham, MA, USA). The maximum projection images were taken with Olympus Fluoview FV3000 confocal laser scanning microscope. Thioflavin-S positive plaque load in subiculum were quantified using NIH Fiji (Image J) software as follows: 1) Duplicate the image under image, which was used the comparison for step 3; 2) Split the channels under image/color/split channers and only keep the green channel image for analysis; 3) Choose Yen to analyze the area (%) of thioflavin-S positive staining under Image/Adjust/Threshold; and 4) Click the measure button to get the value (%) of plaque area in the whole image under Analyze. The average from 6 to 7 sagittal sections per mouse was used to present the plaque load in each mouse's brain.

The numbers of neurons with high intensity of PHF1 or AT8 staining were counted under microscope with 20x objectives, and the sum from 7 sections per animal was used for analysis.

The quantification of Aβ positive staining was conducted as described for Thioflavin S staining.

### 2.18 Human Aβ40 and Aβ42 measurements by ELISA

The level of human Aβ40 and Aβ42 were measured as previous reported (Song et al., 2024). Forty microliter hippocampus homogenate (7.5 %, w/v) was mixed with 60 μl guanidine hydrochloride buffer (50 mM Tris-HCl, pH 8.0, 8.3 M guanidine hydrochloride) 4 h at room temperature. The brain homogenate was further diluted (1:10) with ice-cold PBS containing 1x protease inhibitor cocktail (Calbiochem, cat#539131) and 1 mM AEBSF and centrifuged at 16,000×*g* for 20 min at 4 °C. The supernatant was further diluted 1:4 for Human Aβ40 or 1:128 for Human Aβ42. The Aβ40 and Aβ42 peptide standards were prepared with the same composition of the buffer used for dilution of the samples according to the manufacturer's instructions. The concentration of Human Aβ40 and Human Aβ42 was further normalized to the total protein in the hippocampus homogenate measured by Pierce™ 660 nm protein assay.

### 2.19 Prussian blue staining

The level of microhemorrhages were analyzed by Prussian blue staining as previously reported (Song et al., 2024). Briefly, 6–7 serial sagittal brain sections (40 μm) per mouse were mounted on the slide and dried for overnight at room temperature. Then, the sections was hydrated in distilled water for 3 min and then stained for hemosiderin using 5 % potassium ferrocyanide (ThermoFhishe Scientific, Cat#424130025) in 5 % hydrochloric acid (J.T.Baker, Cat#9535–00) for 15 min. After washing two times with distilled water, the sections were counterstained with nuclear fast red solution (Thermo Scientific, Cat#J61010.AP) for 10 min. The sections were washed twice with distilled water and then dehydrated in 90 % alcohol × 3 min, 95 % alcohol × 3 min, 100 % alcohol for 2 × 3 min. Finally, the slide was cleared with 100 % xylene for 2 × 3 min, followed by a coverslip. The numbers of Prussian blue-positive sites were counted with Olympus Fluoview FV3000 confocal laser scanning microscope under 20 × objective and the average number of sites per section was calculated.

### 2.20 Statistical analysis

Data of biochemical data and behavior tests were analyzed using GraphPad Prism version 5.0 (GraphPad Software Inc, La Jolla, CA, USA) and one-way or two-way ANOVA (as appropriate) followed by a Bonferroni's *posthoc* test. Further intergroup comparisons were also performed using an un-paired two-tailed *t*-test. Paired *t*-test was used for the novel object recognition test. All data are presented as means ± SEM. *P* < 0.05 was considered statistically significant.

## 3 Results

### 3.1 Multi-epitope nanoparticle formulation of the frozen SNAP-AD_5_ vaccine targeting Aβ and tau

**Fig. 1:**
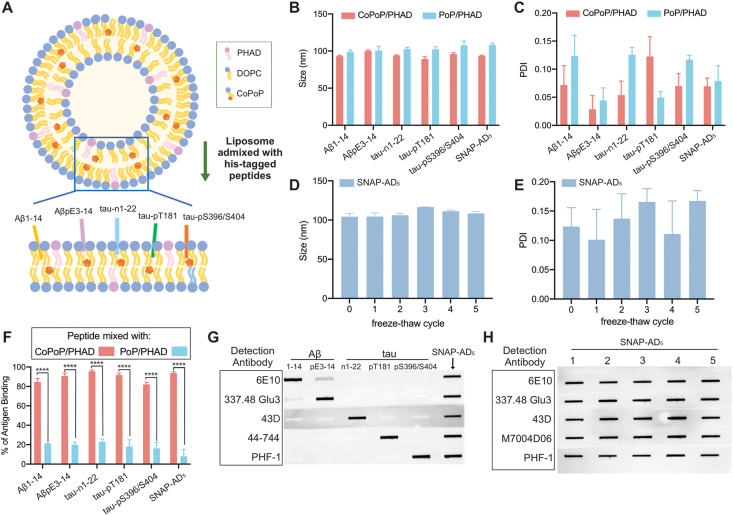
Five his-tagged Aβ/Tau peptides were mixed with CoPoP/PHAD liposomes to form nanoparticles. (A**)** Schematic illustration of SNAP-AD_5_ particle formation with his-tagged antigens, Aβ_1-14_, Aβ_pE3-14,_ tau_n1-22_, tau_pT181_, and tau_pS396/S404_, displayed on CoPoP/PHAD liposome surface. (B) Size, and (C**)** polydispersity of SNAP-AD_5_ were evaluated using dynamic light scattering after a 3-h incubation at 37 °C. (D**)** Size, and (E) polydispersity of SNAP-AD_5_ were assessed using DLS after five freeze-thaw cycles, with each cycle involving a 1-h freeze followed by thawing for measurement. Data are presented as mean ± SD from three independent tests. (F**)** Peptide binding of both individual antigen and SNAP-AD_5_ to CoPoP/PHAD or PoP/PHAD liposomes were measured after 3 h incubation at room temperature using a micro-centrifugal filtration assay. All error bars represent the mean ± SD of triplicate tests. Statistical significance was determined using an unpaired two-tailed *t*-test, with ∗∗∗∗p < 0.0001. (G) Immuno-slot blot assay was used to assess structural integrity and immunoreaction of individual antigen and SNAP-AD_5_ after incorporation into liposomes. (H) Immuno-slot blot assay of SNAP-AD_5_ formulation after five freeze–thaw cycles, probed with Aβ- and Tau epitope–specific antibodies.

Synthetic peptides (Aβ_1-14_, Aβ_pE3-14_, tau_n1-22_, tau_pT181_, and tau_pS396/S404_) were modified with consecutive histidine residues to enable efficient and stable antigen display on the CoPoP/PHAD (CP) liposome surface, as illustrated in Fig. 1A. The insertion of his-tags into CoPoP/PHAD bilayer facilitates a spontaneous nanoliposome antigen particle formation (SNAP) without altering peptide conformation. Both CoPoP/PHAD and PoP/PHAD (2HP) maintained a consistent size of 90–100 nm (Fig. 1B) and polydispersity index (PDI) below 0.5, indicating minimal aggregation after binding with antigens.

To increase stability and facilitate testing and long-term storage, SNAP-AD_5_ was frozen at −80 °C. Upon thawing, similar size (Fig. 1C) and PDI (Fig. 1D) of SNAP-AD_5_ were consistently observed, even across five freeze-thaw cycles. All individual his-tagged peptides efficiently form stable nanoparticles with CoPoP/PHAD liposomes, maintaining slight negative surface charge across all peptide formulation tested (SI Fig. 1). After incubating peptides with liposomes for 3 h at 37 °C, more than 80 % of both individual and multiplexed peptides were converted to particle form, and stably bound to CoPoP liposomes, whereas binding to cobalt-free PoP/PHAD liposomes was inefficient (Fig. 1F). These binding results were also confirmed and consistent with HPLC elution peaks, confirming efficient peptide incorporation into nanoparticle form (SI Fig. 2). In order to verify structural integrity of peptides, an immune-slot blot assay was performed using epitope-specific monoclonal antibodies (mAbs) and polyclonal antibodies (pAbs) (Fig. 1G**).** All antibody reactivity was maintained throughout multiple freeze-thaw cycles (Fig. 1H). Each individual peptide displayed strong reactivity to its specific antibodies, without detectable cross-reactivity. Aβ_pE3-14_ showed lower reactivity with the 6E10 antibody, likely due to its sequence similarity with Aβ_1-14_, apart from a pyroglutamate modification at the third amino acid (Fig. 1G). The SNAP-AD_5_ vaccine formulation was successfully detected by all antibodies under both conditions (1) following 3 h incubation at 37 °C, and (2) after undergoing five freeze-thaw cycles, confirming that each antigen remained stably bound to the liposomes with accessible epitope sites. Overall, the SNAP-AD_5_ vaccine formulation retained stability and antigen accessibility across varying conditions, demonstrating its potential as a candidate for immunogenic applications.

### 3.2 Antibody response of SNAP-AD_5_ with immunization

**Fig. 2:**
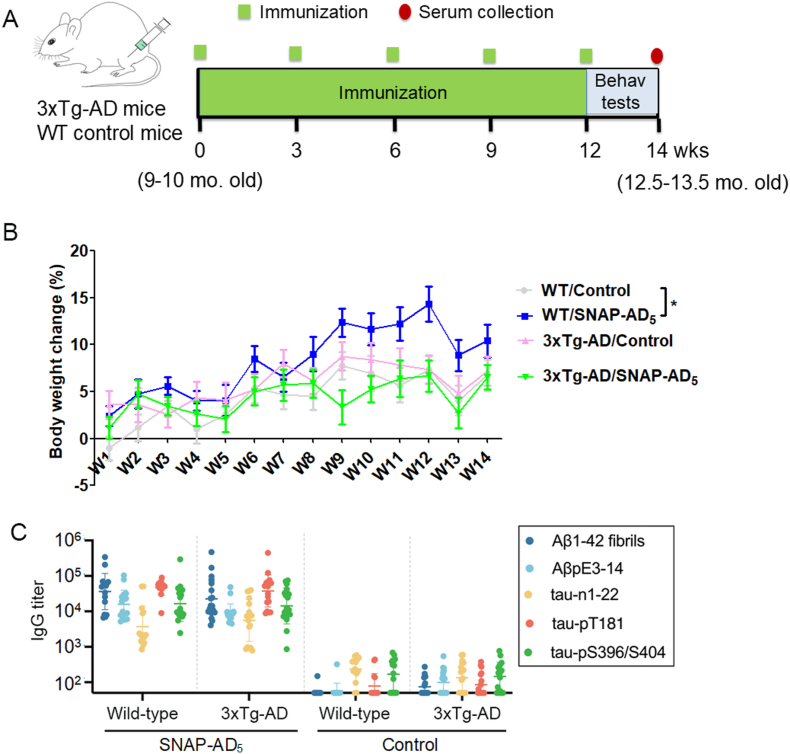
Study design and timeline, body weight and antibody titers. (A) 3xTg-AD and wildtype (WT) control mice at 9–10 months old were immunized with SNAP-AD_5_ vaccine or control (without antigen) for 5 doses with a 3-week interval. Behavioral (Behav) tests, including open field, one-trial novel object recognition test and Morris water Maze test, were conducted 3 days after the 5th immunization. All animals were euthanized after behavioral tests. (B) Body weight changes (%) were recorded once a week during the whole period of experiment. ∗*p <* 0.05 by ANOVA followed by a Bonferroni's *posthoc* test. (C) The antibody titers accessed by ELISA coating with Aβ_1-42_ fibrils, Aβ_pE3-14_, tau_1-22_, tau_pT181_, tau_pS396/S404_ 15 days after the 5th immunization. Error bars show mean ± SEM. N = 15 for WT/Control, 16 for WT/SNAP-AD_5_, 17 for 3xTg-AD/Control and 17 for 3xTg-AD/SNAP-AD_5_. Statistical analysis of IgG titers was performed between WT/3xTg-AD vaccinated with SNAP-AD_5_ and WT/3xTg-AD untreated by one-way ANOVA followed by Tukey's test using log-transformed values**, *∗∗∗∗p < 0.0001 (not shown in figure)*.**

Since 3 × Tg-AD mice display learning and memory deficits starting at 6.5 months of age (Stover et al., 2015), develop amyloid plaques starting at about 9 months of age and NFTs starting about 12 months of age (Oddo et al., 2003), we started to immunize the 9-10-month-old 3xTg-AD mice and age-matched WT mice for 5 immunizations with a 3-week interval to test the therapeutic activity of SNAP-AD_5_. Behavioral tests, including open field test, one-trial novel object recognition test and Morris water maze test, were performed starting 3 days after the last doses of immunization (Fig. 2A). Immunization with SNAP-AD_5_ did not cause significant body weight changes in 3xTg-AD mice, but the mice in the group of WT/SNAP-AD_5_ exbited slightly more bodyweight gain from week 9 than the mice in the WT/Control group (Fig. 2B). Additionally, we also monitored the general conditions of the mice during the whole period of study. We did not observe any noticeable changes in general health conditions in the animals. These results indicate that immunization with SNAP-AD_5_ does not result in adverse side effects. Importantly, immunization with SNAP-AD_5_ generated high antibody titers against its specific antigens in both WT and 3xTg-AD mice. Each antigen induced similar levels of antibody titers in WT and 3xTg-AD mice (Fig. 2C). These results indicate that the SNAP-AD_5_ vaccine maintains high immunogenicity and produces high levels of specific antibodies in mice.

### 3.3 SNAP-AD_5_ improves cognitive function in 3xTg-AD mice

**Fig. 3:**
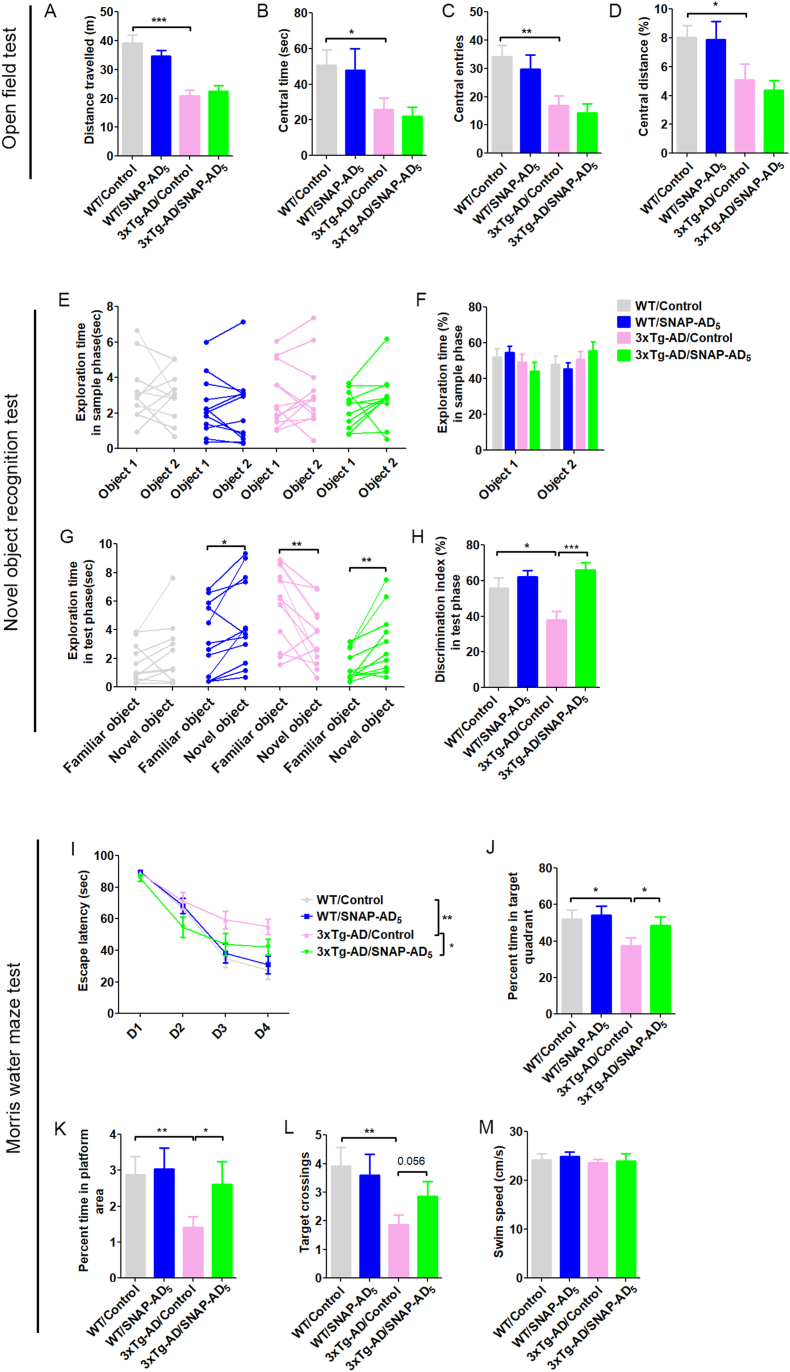
Immunization with SNAP-AD_5_ improves cognitive function in 3xTg-AD mice**.** (A–D) Open field test. (A) Total distance travelled in apparatus; (B) Central area time (sec); (C) Central area entries; (D) Percent distance travelled in central area. N = 15 for WT/Control, 16 for WT/SNAP-AD_5_, 17 for 3xTg-AD/Control, and 17 for 3xTg-AD/SNAP-AD_5_ for open field test. ∗*p <* 0.05, ∗∗*p <* 0.01 and ∗∗∗*p <* 0.001 by ANOVA followed by a Bonferroni's *posthoc* test. (E–H) One trial novel object recognition test. (E) The time spent exploring two identical object 1 and object 2 in sample phase. (F) The percentage of time spent exploring two identical objects during sample phase; (G) The time spent exploring novel object and familiar object during test phase. (H) Discrimination index. N = 11 for WT/Control, 12 for WT/SNAP-AD_5_, 12 for 3xTg-AD/Control, and 11 for 3xTg-AD/SNAP-AD_5_ for novel object recognition test. (E and G), ∗*p <* 0.05 and ∗∗*p <* 0.01 by paired *t*-test, (F and H). ∗*p <* 0.05 and ∗∗∗*p <* 0.001 by ANOVA followed by a Bonferroni's *posthoc* test. (I–M) Morris water maze test. (I) Escape latency (sec) to reach the hidden platform during acquisition phase for 4 consecutive days; (J) Percent time in the target quadrant; (K) Percent time in the platform area; (L) Number of target crossings; (M) Swim speed during the probe trial. N = 11 for WT/Control, 15 for WT/SNAP-AD_5_, 15 for 3xTg-AD/Control, and 14 for 3xTg-AD/SNAP-AD_5_ for Morris water maze test. ∗*p <* 0.05, ∗∗*p <* 0.01 and ∗∗∗*p <* 0.001 by ANOVA followed by a Bonferroni's *posthoc* test.

3xTg-AD mice develop cognitive impairment as early as 6 month olds (Stevens and Brown, 2015). To investigate whether SNAP-AD_5_ could rescue cognitive impairment in 3xTg-AD mice, serial behavioral tests from less stressful to more stressful tests were performed. First, we performed open field test to investigate whether there are any differences in locomotive activity and anxiety, which could potentially affect the interpretation of cognitive tests (Mah et al., 2016; Scott et al., 2015). We found that 3xTg-AD mice exhibited lower locomotive activity, as evidenced by less total travelled distance (Fig. 3A), and more anxiety, as evidenced by less time in central area (Fig. 3B), less number of central area entries (Fig. 3C) and lower percentage of central area distance (Fig. 3D) than the WT mice (Fig. 3A), suggesting that the 3xTg-AD mice developed some level of anxiety at this age. Nevertheless, vaccination with SNAP-AD_5_ did not make any significant difference in total travelled distance, central time, number of central entries, or percent central area distance in the central area in either WT mice or 3xTg-AD mice (Fig. 3A–D). These results indicate that the SNAP-AD_5_ vaccine did not affect the locomotive activity or anxiety level in mice.

We performed one-trial novel object recognition test to assess whether SNAP-AD_5_ could improve short-term memory. All the mice in four groups spent similar time on two identical objects during the 5-min sample phase (Fig. 3E and F), suggesting that the animals did not exhibit location bias. During the test phase, the WT mice with and without the SNAP-AD_5_ immunization both showed clear discrimination between the novel and familiar objects, indicating a lack of significant impact of SNAP-AD_5_ on short-term memory and also the validity of the test performed (Fig. 3G and H). However, the 3xTg-AD/Control mice spent somewhat less time on the novel object than the familiar object, indicating a clear short-term memory deficit of these mice, as expected. We observed that, like the WT mice, the 3xTg-AD/SNAP-AD_5_ mice spent much more time on exploring the novel object than the familiar one (Fig. 3G and H). These results demonstrate that vaccination with SNAP-AD_5_ can rescue short-term memory deficit in 3xTg-AD mice.

To further assess the effect of SNAP-AD_5_ on the cognitive function, we performed Morris water maze test to assess whether SNAP-AD_5_ can rescue spatial learning and memory impairment in 3xTg-AD mice. Compared with the mice in group of WT/Control, the mice in group of 3xTg-AD/Control took longer time to find the hidden platform during the 4-day acquisition phase (Fig. 3I) and spent less time in the target quadrant (Fig. 3J) and the platform area (Fig. 3K), and less numbers of target crossings (Fig. 3L) during the probe test. These results indicate that 3xTg-AD mice had impaired spatial learning and memory at tested age. However, compared with the mice in group 3xTg-AD/Control, the mice in group of 3xTg-AD/SNAP-AD_5_ took less time to find the hidden platform (Fig. 3I) during acquisition phase, and spent more time in the target quadrant (Fig. 3J) and in the platform location (Fig. 3K), and more numbers of target crossings than the mice in group of 3xTg-AD/Control (Fig. 3L). The performance of the 3xTg-AD mice after immunization was indistinguishable from the WT control mice. These results indicate that SNAP-AD_5_ can rescue the impairment of spatial learning and memory in 3xTg-AD mice. Vaccination with SNAP-AD_5_ did not impact the spatial learning and memory in WT mice (Fig. 3I–L). There were no significant differences in the swimming speeds among the groups (Fig. 3M).

### 3.4 SNAP-AD_5_ decreases Aβ accumulation in 3xTg-AD mice

**Fig. 4:**
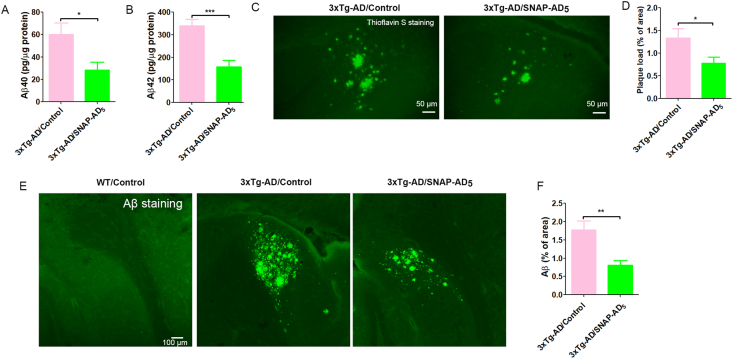
Immunization with SNAP-AD_5_ reduce Aβ pathology. The level of Aβ40 (A) and Aβ42 (B) in hippocampus was measured by ELISA. (C) Thioflavin S staining of Aβ plaque in the subiculum area of 3xTg-AD mouse brains. (D) Percent of thioflavin S positive plaque area from 6 to 7 sections (every 6th sagittal brain section per mouse). (E) Aβ staining (Cat#8243, Cell Signaling) in the subiculum area of 3xTg-AD mouse brains. (F) Percent of Aβ positive staining from 6 to 7 sections (every 6th sagittal brain section per mouse). Data are presented as mean ± SEM. ∗*p <* 0.05, ∗∗*p <* 0.01, and ∗∗∗*p <* 0.001 by unpaired two-tailed *t*-test. N = 17 for 3xTg-AD/Control, and 17 for 3xTg-AD/SNAP-AD_5_.

The 3xTg-AD mice had already developed amyloid plaques when we initiated the immunization with SNAP-AD_5_ at 9–10 months of age (Belfiore et al., 2019). We first analyzed the levels of human Aβ40 and Aβ42 in the hippocampus of the 3xTg-AD mice immunized with or without SNAP-AD_5_. We found that SNAP-AD_5_ treatment decreased the levels of both Aβ40 and Aβ42 (Fig. 4A and B). Thioflavin S staining, which stains β-sheet-rich structure in Aβ plaques, confirmed that SNAP-AD_5_ treatment dramatically decreased the area of thioflavin S positive staining in the subiculum area of the hippocampus (Fig. 4C and D). The results of Aβ staining further demonstrated that SNAP-AD_5_ can reduce the Aβ pathology (Fig. 4E and F). Collectively, these results demonstrate that the SNAP-AD_5_ vaccine can decrease Aβ accumulation in the mouse brains.

### 3.5 SNAP-AD_5_ decreases tau pathology in 3xTg-AD mice

**Fig. 5:**
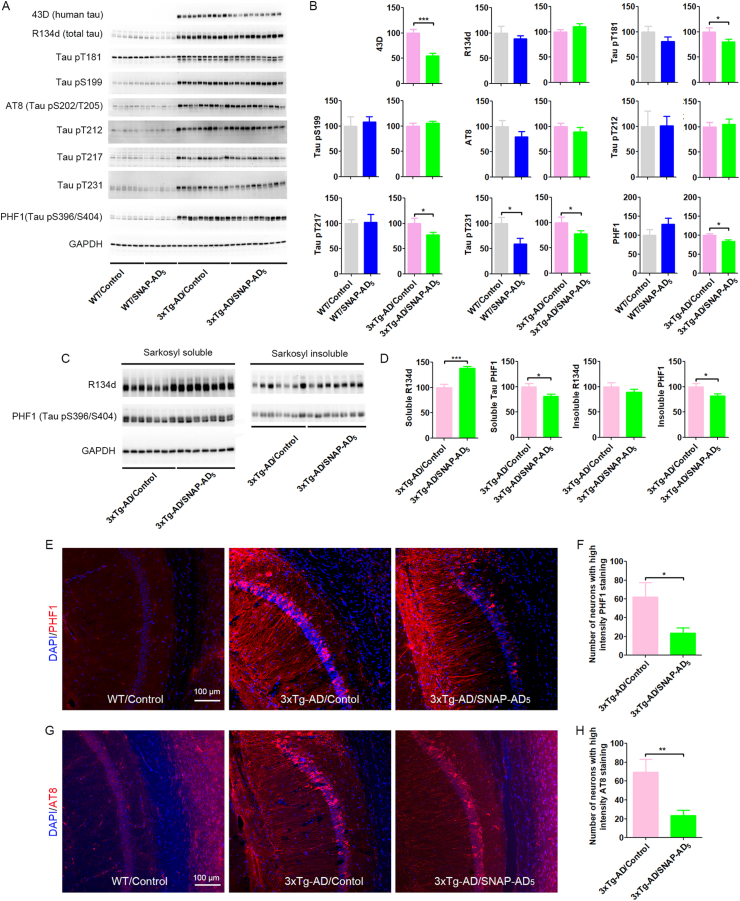
Immunization with SNAP-AD_5_ decreased tau pathology. (A) Representative Western blots of the hippocampus developed with 43D against human transgenic tau, R134d against total tau, and with several phosphorylation-dependent and site-specific tau antibodies. (B) Densitometric quantification of the blots in A after normalization with total tau level (R134d blot). ∗*p <* 0.05, ∗∗∗*p <* 0.001 by unpaired two-tailed *t*-test. N = 6 for WT**/**Control, 6 for WT/SNAP-AD_5_, 10 for 3xTg-AD/Control, and 10 for 3xTg-AD/SNAP-AD_5_. (C) Representative Western blots of sarkosyl-soluble and sarkosyl-insolube fractions of the cerebrocortex developed with R134d against total tau and PHF1 against tau phosphorylation at Ser396/Ser404 sites. (D) Densitometric quantification of the blots shown in C after normalization with sarkosyl-soluble tau level (R134d blot). N = 7 for 3xTg-AD/Control, and 7 for 3xTg-AD/SNAP-AD_5_. Data are the percentage of control-treated animals and are presented as mean ± SEM. ∗*p <* 0.05, ∗∗∗*p <* 0.001 by unpaired two-tailed *t-test*. Immunostaining with PHF1 antibody (E) and AT8 antibody (G) of the CA1 and subiculum sectors of the hippocampus of 3xTg-AD mice with or without prior immunization with the SNAP-AD_5_ vaccine. The section from group of WT/control was used as control. The sum of neurons with high intensity PHF1 staining (F) and AT8 staining (H) from 7 serial sections (every 6th sagittal brain section) per mouse. N = 17 for 3xTg-AD/control, and 17 for 3xTg-AD/SNAP-AD_5_. Data are shown as mean ± SEM. ∗∗*p <* 0.01 by unpaired two-tailed *t-test*.

We measured the protein levels of total tau, transgenic human tau, and hyperphosphorylated tau at multiple sites to determine the effect of SNAP-AD_5_ on tau pathology in the hippocampus. We found that the SNAP-AD_5_ significantly decreased the 43D level, which only recognizes human tau 6–18 (Fig. 5A and B). The levels of total tau tested by R134d antibody did not exhibit significant difference between the control (SNAP only) and SNAP-AD_5_ groups in either WT or 3xTg-AD mice (Fig. 5A and B). Vaccination with SNAP-AD_5_ decreased tau phosphorylation at Thr181, Thr217, Thr231, and Ser396/404, but did not change the level of phosphorylated tau at Ser199, Thr202/205, or Thr212 (Fig. 5A and B) tested by Western blots.

We also found immunization with SNAP-AD_5_ significantly increase sarkosyl soluble tau level tested by R134d antibody in the cortex of 3xTg-AD mice (Fig. 5C and D), which was consistent with our previous study (Song et al., 2024). Quantifications of tau phosphorylation levels at Ser396/Ser404 sites after normalization with the total tau level indicated that SNAP-AD_5_ vaccine reduced tau phosphorylation in the cortex, as observed in the hippocampus in 3xTg-AD mice (Fig. 5Cand D). Importantly, SNAP-AD_5_ vaccine also significantly reduced the level of sarkosyl insoluble tau phosphorylation at Ser496/404 sites (Fig. 5C and D).

We further employed immunohistochemical studies to explore whether SNAP-AD_5_ vaccine could reduce tau pathology. We observed a clear reduction of neurons with high intensity PHF1 (Fig. 5E and F) in the brains of 3xTg-AD mice immunized with SNAP-AD_5_ vaccine compared to those without immunization. Similar results were also observed with AT8 staining (Fig. 5G and H). Collectively, these results indicate that SNAP-AD_5_ vaccine can decrease tau pathology.

### 3.6 SNAP-AD_5_ does not cause inflammation response in the mouse brain or in the peripheral system

**Fig. 6:**
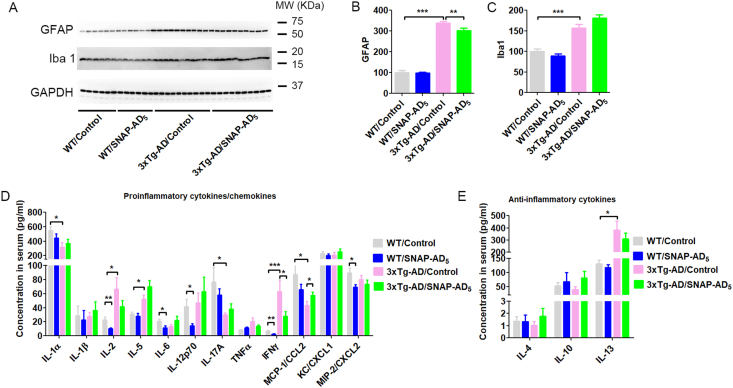
Immunization with SNAP-AD_5_ did not induce inflammation response in mouse brain or in peripheral circulation systems. (A) Representative immunoblots of GFAP and Iba 1 in the hippocampus. Densitometric quantification of the blots of GFAP (B) and Iba 1 (C) in A. ∗∗*p <* 0.01 and ∗∗∗*p <* 0.001 by ANOVA followed by a Bonferroni's *posthoc* test N = 6 for WT/Control, 6 for WT/SNAP-AD_5_, 10 for 3xTg-AD/Control, and 10 for 3xTg-AD/SNAP-AD_5_. (D) The levels of proinflammatory cytokines or chemokines in plasm. (E) The levels of anti-inflammatory cytokines in plasm. ∗*p <* 0.05, ∗∗*p <* 0.01 and ∗∗∗*p <* 0.001 by ANOVA followed by a Bonferroni's *posthoc* test. Sample sizes for each cytokine or chemokine were listed at Supplementary Table 1.

To investigate whether immunization with SNAP-AD_5_ could cause neuroinflammation, we first investigate the levels of calcium binding adaptor molecule 1 (Iba1) as the marker of microglia, and glial fibrillary acidic protein (GFAP) as the marker of astrocyte in brain. We found that 3xTg-AD mice had higher levels of Iba 1 and GFAP than WT mice (Fig. 6A–C), suggesting that 3xTg-AD mice exhibited neuronal inflammation, which is consistent to previous reports (Belfiore et al., 2019). Immunization with SNAP-AD_5_ did not increase the brain levels of either Iba1 or GFAP in mice. Instead, it decreased GFAP level slightly in the 3xTg-AD mice. These results suggest that SNAP-AD_5_ did not induce neuroinflammation in the mouse brain.

We further analyzed the proinflammatory cytokines/chemokines and anti-inflammatory cytokines in the peripheral plasma. We found significant alterations of the cytokine levels in the plasma of 3xTg-AD mice as compared to the WT control mice, suggesting some disturbance of the peripheral immune system of the 3xTg-AD mice. These changes increased levels of IL-2, IL-5, IFNγ and IL13, and decreased levels of IL-1α, IL-17A and MCP-1/CCL2 (Fig. 6D and E). Interestingly, vaccination with SNAP-AD_5_ did not lead to increases in any proinflammatory cytokines/chemokines in either WT mice or 3xTg-AD mice except for a slight increase in the MCP-1/CCL2 level in the 3xTg-AD mice. Surprisingly, vaccination with SNAP-AD_5_ downregulated proinflammatory cytokines/chemokines of IL-2, IL-6, IL-12P70, IFNγ and MIP-2/CXCL2 in WT mice, and IFNγ in 3xTg-AD mice (Fig. 6D). These results suggest that immunization with SNAP-AD_5_ did not induce peripheral inflammation responses.

**Fig. 7:**
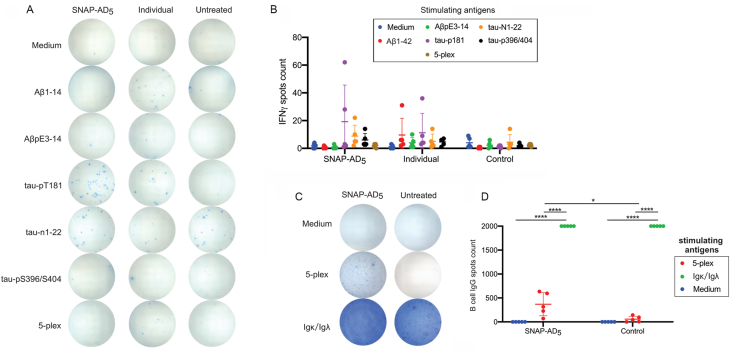
SNAP-AD_5_ immunization elicited antigen-specific B cell response without high antigen-specific T cell response in mice. (A) Representative ELISPOT wells showing IFN-γ–secreting splenocytes from SNAP-AD_5_ immunized, individual antigen immunized, or untreated control mice following stimulation with medium, individual antigens, or a 5-plex antigen mix. (B) Quantification of IFN-γ spots from (A) were counted and analyzed by a two-way ANOVA followed by Bonferroni's multiple comparison test, comparing each experimental group to the control, ∗p < 0.05. (C) Representative ELISPOT wells showed B-cell producing IgG in blood from mice (n = 5) immunized with SNAP-AD_5_ or untreated mice. Blood cells after lysis were stimulated with medium alone, 10 μg/mL of Igκ/Igλ antibodies, or 10 μg/mL 5-plex mixed antigen. (D) Quantification of B-cell producing IgG spots from (C) were analyzed by a one-way ANOVA with multiple comparison followed by Tukey's test, ∗*P<0.05, ∗∗∗∗P<0.0001*.

To further characterize the immune response elicited by SNAP-AD_5_, we performed ELISPOT assays to examine T-cell and B-cell activity. The representative ELISPOT results showed that SNAP-AD_5_ immunization did not induce high antigen-specific IFN-γ–secreting T cells (Fig. 7A and B), consistent with the absence of T-cell–driven proinflammatory cytokine responses. In contrast, SNAP-AD_5_ immunization elicited antigen-specific B-cell responses, as evidenced by increased numbers of IgG-producing B cells following stimulation with the 5-plex antigen mix or anti-Igκ/Igλ antibodies compared to untreated controls (Fig. 7C and D). For wells coated with anti-Igκ/Igλ antibodies, spot counts exceeded the upper detection limit of the ELISPOT reader; therefore, values were capped at 2000 spots/well for analysis. These findings indicate that SNAP-AD_5_ vaccination preferentially promotes B-cell–mediated immunity without activating proinflammatory T-cell responses.

### 3.7 SNAP-AD_5_ reduces microhemorrhages in the brain of 3xTg-AD mice

**Fig. 8:**
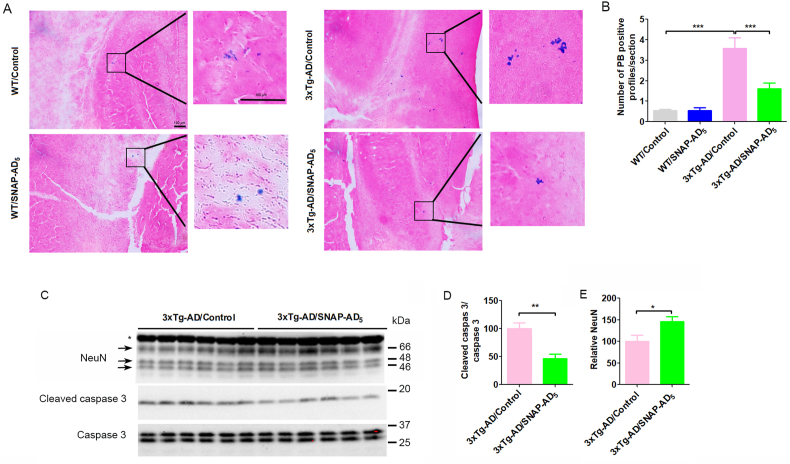
Immunization with SNAP-AD_5_ decreases microhemorrhages in the brain of 3xTg-AD. (A) Representative image for Prussian blue staining in WT/Control, WT/SNAP-AD_5_, 3xTg-AD/Control, and 3xTg-AD/SNAP-AD_5_ mice. (B) Quantification of Prussian blue positive profiles. The Prussian blue positive profiles were counted from 5 to 7 sections which were from every 6th sagittal brain section per mouse. N = 14 for WT/Control, 16 for WT/SNAP-AD_5_, 17 for 3xTg-AD/Control, and 17 for 3xTg-AD/SNAP-AD_5_. *∗∗∗p < 0.*001 by ANOVA followed by a Bonferroni's posthoc test. (C) Representative Western blots of the hippocampal tissue developed with total caspase 3 and cleaved caspase 3. The membrane for cleaved caspase 3 was reblotted with NeuN (Clone A60). ∗Cross band with synapsin 1. (D) Densitometric quantification of cleaved caspase 3 in C after normalization with total caspase 3. N = 6 for 3xTg-AD/Control, and 6 for 3xTg-AD/SNAP-AD_5_. ∗∗*p <* 0.01 by unpaired two-tailed *t*-test. (E) Densitometric quantification of NeuN at 46-, 48-, and 66-kDa bands. N = 6 for 3xTg-AD/Control, and 6 for 3xTg-AD/SNAP-AD_5_. ∗*p <* 0.05 by unpaired two-tailed *t*-test. (For interpretation of the references to color in this figure legend, the reader is referred to the Web version of this article.)

Since Aβ immunotherapy can induce microhemorrhages in the brain, we investigated the level of microhemorrhages by Prussian blue (PB) staining to determine whether immunization of mice with SNAP-AD_5_ induces microhemorrhages. As expected, almost no PB staining was observed in the brain sections of WT mice, including those after immunization with SNAP-AD_5_ (Fig. 8A and B). A small number of PB staining were seen in the brains of 3xTg-AD mice treated with control SNAP, suggesting a few microhemorrhages present in the 3xTg-AD mouse brains at the age of the study (Fig. 8B). Surprisingly, we found significantly decreased number of PB staining in the brains of 3xTg-AD mice after SNAP-AD_5_ immunization as compared to the 3xTg-AD/Control mice. These results suggest that the SNAP-AD_5_ vaccine does not induce microhemorrhages in WT mouse brains and might instead prevent them in the brains of 3xTg-AD mice.

### 3.8 SNAP-AD_5_ reduces apoptosis in the brain of 3xTg-AD mice

Aβ and tau pathologies in 3xTg-AD mice may trigger brain cell apoptosis, but overexpression of Bcl-2, an anti-apoptotic protein, could reduce AD-like pathologies in the brain of 3xTg-AD mice (Rohn et al., 2008). Since SNAP-AD_5_ reduced both Aβ and tau pathologies, we investigated whether immunization with SNAP-AD_5_ can reduce apoptosis. We observed that immunization with SNAP-AD_5_ vaccine significantly reduced the level of cleaved caspase 3 (Fig. 8C and D). Consistently, SNAP-AD_5_ vaccine also increased the protein level of NeuN, a neuronal marker (Fig. 8C and E). These results suggest that vaccination with SNAP-AD_5_ may reduce cellular apoptosis and prevent or rescue neuronal death in the brains of 3xTg-AD mice.

## 4 Discussion

Developing clinically efficacious therapeutic approaches for treating AD is an unmet clinic need because of the unsatisfactory outcomes of current available treatments. FDA approved Aβ monoclonal antibodies, including aducanumab, lecanemab and donanemab, only slightly slow down the rate of cognitive decline in patients with early stage of AD, but could not rescue the cognitive impairment in AD patients. Tau immunotherapy for treating AD is still in the early stage of development, and so far no promising outcomes have been released from clinical trials. Since the synergistic effect of Aβ and tau proteins in driving the pathogenesis and progression of AD, developing an approach simultaneously reducing both Aβ and tau pathologies would be more effective than the current monotherapies targeting either Aβ or tau protein. We have developed a novel vaccine harboring a mixture of his-tagged Aβ_1-14_, Aβ_pE3-14_, tau_pT181_, tau_pT217_, and tau_pS396/S404_ peptides. The antibodies produced from this vaccine not only recognizes Aβ plaques and NFTs of human AD brains, but also demonstrates potent prophylactic activity in 3xTg-AD mice (Song et al., 2024).

In the present study, we modified the vaccine using tau_1-22_ peptide instead of tau_pT217_ and tested its therapeutic effect. We found that this SNAP-AD_5_ vaccine produced high titers of antibody against its specific antigens, reduced both Aβ and tau pathologies, and rescued cognitive impairment. Importantly, this vaccine was well tolerated and did not induce inflammation response in the brain or in the periphery. Furthermore, the immunization appeared to reduce microhemorrhages in 3xTg-AD mice.

Developing a potent vaccine for clinical use should harness the immune system to trigger the immunogenicity without activation of harmful T cells response. Activation of cytotoxic T cells leads to the release of pro-inflammatory cytokines, such as IFN-γ, TNF-α, and IL-2, which can cause severe and unpredictable adverse effects such as the meningoencephalitis observed in patients immunized with AN1792, the first full-length Aβ vaccine, which contained the Aβ16-33 T-cell epitope (Monsonego et al., 2003; Orgogozo et al., 2003). To avoid the T-cell response caused by the central part of full-length Aβ, active vaccine with partial sequences selected from Aβ1-14 facilitated B-cell receptor binding onto a B cell surface, which triggers intracellular signaling pathways and strong B-cell activation without T-cell help in order to induce immune response (Winblad et al., 2012). Tau immunotherapies currently under development are intentionally designed to elicit B-cell responses, supported when necessary, by helper T-cell activity, while minimizing cytotoxic CD8^+^ T-cell engagement. Consistent with this design principle, no evidence of gliosis, T-cell activation, or other inflammatory markers has been reported in preclinical or clinical studies of tau immunotherapies (Sandusky-Beltran and Sigurdsson, 2020; Sigurdsson, 2008). Therefore, the SNAP-AD_5_ vaccine incorporates two Aβ peptides and three tau peptides selected to avoid induction of cytotoxic T-cell responses. In our previous prophylactic study, immunization with our vaccine did not result in activation of T cell response with each peptide. Immunization with the vaccine did not cause peripheral inflammatory response which was analyzed at 30 weeks after last dose (4th) immunization, and even decreased the levels of IL-6, IFNγ, GM-CSF, and IL-12p70 in 3xTg-AD mice, which suggests that immunization with SNAP-AD_5_ not only did not cause inflammatory response in long-term, but also inhibited the inflammation (Song et al., 2024). In the present therapeutic study, we analyzed the inflammatory cytokines 2 weeks after last dose of immunization, which represents the acute phase response to the immunization. Consistent with previous prophylactic study, 5-doses of immunization with our SNAP-AD_5_ vaccine did not elicit detectable T-cell activation (Fig. 6D; Fig. 7A and B), which indicates that SNAP-AD_5_ does not induce a robust pro-inflammatory Th1-type T-cell response. Interestingly, immunization with SNAP-AD_5_ did not activate the microglia cells in the brain, or even decrease the astrocytes in the brain and reduced serum IFN-γ levels, which may reflect decreased Aβ and tau pathologies which can drive deleterious T-cell activation. Altogether, these data indicate that SNAP-AD_5_ vaccine does not cause any detectable acute or chronic inflammatory response in 3xTg-AD mice.

The major side effect of the FDA-approved Aβ immunotherapy is the high incidence of amyloid-related imaging abnormalities (ARIA) including cerebral microhemorrhages, especially in the patients with APOE-e4 allele. The mechanisms underlying these potentially serious adverse events have not been fully elucidated. In the present study, we found the SNAP-AD_5_ vaccine might reduce microhemorrhages in 3xTg-AD mice and did not induce the microhemorrhages in WT mice, which is consistent with results in the previous prophylactic study (Song et al., 2024). Reduction of Aβ pathology may contribute to the less microhemorrhages in the vaccinated 3xTg-AD mice. These results suggest that our SNAP-AD_5_ vaccine is safe and unlikely cause microhemorrhages in both short-term and long-term after immunization.

The present study employed 3xTg-AD mice at an early stage of AD-like brain pathologies and cognitive impairment. Whether the efficacy of our vaccine can be also observed in older mice remains to be studied. One limitation of the present study is that only female mice were used. Given that some sex-dependent differences have been observed in 3xTg-AD mice (Barber et al., 2024; Dennison et al., 2021), future studies will test the therapeutic efficacy of SNAP-AD_5_ vaccine in male 3xTg-AD mice. The SNAP-AD vaccine has shown prolonged effects lasting at least 31 weeks after completion of vaccination in reducing Aβ and tau pathologies in a prophylactic study in which female 3xTg-AD mice were vaccinated prior to the onset of pathology (Song et al., 2024). Together with the findings from the current therapeutic study showing that SNAP-AD_5_ markedly reduced Aβ and tau pathologies when vaccination was initiated after pathology had already developed in female 3xTg-AD mice, we anticipate similar beneficial outcomes in male 3xTg-AD mice since male 3xTg-AD mice typically exhibit lower levels of Aβ and tau pathology than females (Barber et al., 2024). Additionally, no single mouse model can fully replicate AD pathologies occurred in human AD brain. Therefore, future studies in other mouse models will be performed to validate the therapeutic potential of SNAP-AD_5_ vaccine. Positive outcomes would improve prospects for clinical application.

At present, the clinical outcomes including the side effects from a phase II/III combinational immunotherapy with lecanemab and E2814 in patients with early onset AD are still unavailable. The vaccines targeting dual Aβ and tau either only demonstrated the reduction of Aβ and/or tau pathologies without the data on cognitive function (Barbour et al., 2024; Davtyan et al., 2019), or did not report the data on safety (Barbour et al., 2024; Davtyan et al., 2019; Feng et al., 2024). Our SNAP-AD_5_ vaccine incorporates a multivalent design, enabling simultaneous targeting of both pathological hallmarks of AD. The dual-targeting strategy enhances a broader immune response. In addition, the use of liposomal platform contributes to a safety profile by minimizing off-target T cell activation while maintaining strong immunogenicity. Therefore, these features highlight SNAP-AD_5_ as a potential approach in reducing Aβ and tau, and in improving cognitive function.

## 5 Conclusions

Overall, the SNAP-AD_5_ vaccine produced high antibody titers against its specific antigens in both WT and 3xTg-AD mice. Immunization with SNAP-AD_5_ vaccine demonstrated its potential therapeutic activity in reduction of both Aβ and tau pathologies, improvement of cognitive function, and did not induce neuroinflammation or cerebral microhemorrhages. Together with our recent prophylactic study, our findings support further development of the SNAP-AD_5_ vaccine as a novel vaccine for preventing and/or treating AD.

## CRediT authorship contribution statement

**Chun-Ling Dai:** Writing – review & editing, Writing – original draft, Methodology, Investigation, Funding acquisition, Formal analysis, Data curation, Conceptualization. **Yiting Song:** Writing – review & editing, Writing – original draft, Methodology, Investigation, Formal analysis, Data curation. **Yonghua Chen:** Formal analysis. **Yunn Chyn Tung:** Formal analysis. **Wei-Chiao Huang:** Funding acquisition, Formal analysis. **Cheng-Xin Gong:** Writing – review & editing, Writing – original draft, Supervision, Project administration, Formal analysis, Data curation, Conceptualization. **Jonathan F. Lovell:** Writing – review & editing, Writing – original draft, Project administration, Investigation, Formal analysis, Data curation, Conceptualization.

## Availability of data and materials

Data will be made available on request.

## Funding

This work is supported by National Institute on Aging, 10.13039/100015412Small Business Technology Transfer (STTR) grant [Grant number R41AG082620, R42AG082620] to Chun-Ling Dai and Wei-Chiao Huang. This work is supported in part by the New York State Office for People with Developmental Disabilities to Chun-Ling Dai.

## Declaration of competing interest

Jonathan F. Lovell and Wei-Chiao Huang hold interest in POP Biotechnologies. Wei-Chiao Huang is an employee of POP Biotechnologies. Cheng-Xin Gong serves as a consultant to POP Biotechnologies, Inc., Alectos, Inc., and Phanes Biotech, Inc. Other authors declare that they have no known competing financial interests or personal relationships that could have appeared to influence the work reported in this paper.

## Data Availability

Data will be made available on request.

## Abbreviations

AD: Alzheimer's disease; Aβ: Amyloid beta; NFTs: Neurofibrillary tangles; Triple transgenic AD mouse: 3xTg-AD mouse; WT: Wild type; SNAP: spontaneous nanoliposome antigen particle; DLS: dynamic light scattering.
