# Supplementary material for: Therapeutic nanoliposome vaccine targeting multiple Aβ and tau epitopes reduces AD-like brain pathologies and rescues cognitive deficits in 3xTg-AD mice
Source: Brain Behav Immun Health. 2025 Dec 30;51:101167. doi: 10.1016/j.bbih.2025.101167 (PMC12813361; doi:10.1016/j.bbih.2025.101167)
Supplement: Multimedia component 1 [file mmc1.docx]

Supporting Information

**Therapeutic nanoliposome vaccine targeting multiple Aβ and tau epitopes reduces AD-like brain pathologies and rescues cognitive deficits in 3xTg-AD mice**

Chun-Ling Dai^a,#,1^, Yiting Song^b,1^, Yonghua Chen^a^, Yunn Chyn Tung^a^, Wei-Chiao Huang^b,c^, Cheng-Xin Gong^a^, Jonathan F. Lovell^b,#^

^1^Equal contribution

^#^Correspondence: chunling.dai@csi.cuny.edu; jflovell@buffalo.edu

^a^ Department of Neurochemistry, Inge Grundke-Iqbal Research Floor, New York State Institute

for Basic Research in Developmental Disabilities, Staten Island, NY 10314, USA

^b^ Department of Biomedical Engineering, State University of New York at Buffalo, Buffalo,

NY 14260, USA

^c^ POP Biotechnologies, Buffalo, NY 14228, USA


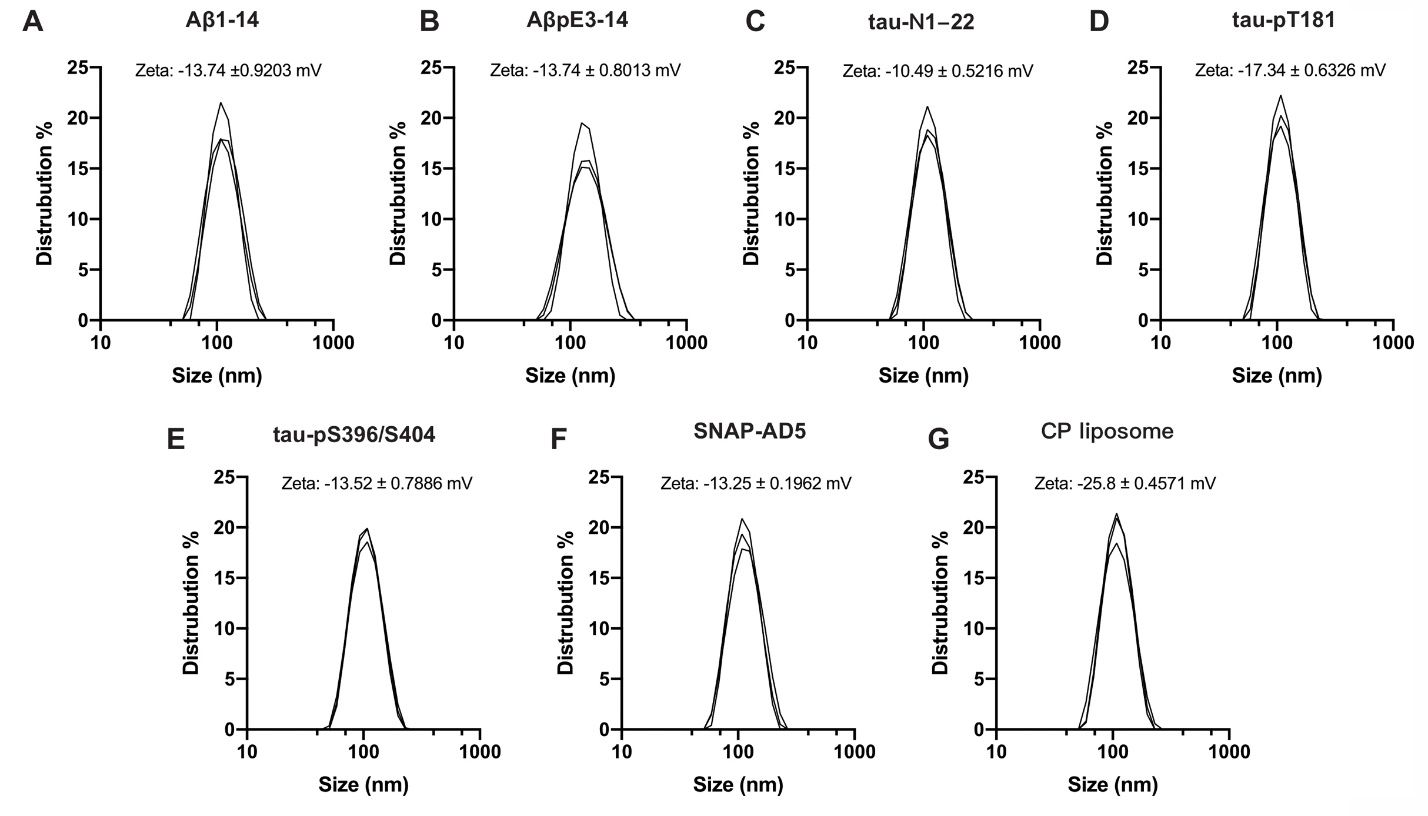


SI Figure 1: Zeta potential and dynamic light scattering analysis of individual liposomal peptide, SNAP-AD_5_, and control liposome. Dynamic light scattering analysis was performed for liposomal peptides, (A) Aβ1-14, (B) AβpE3-14, (C) tau-N1-22, (D) tau-pT181, (E) tau-pS396/S404, (F) SNAP-AD_5_, and (G) CoPoP/PHAD (CP) liposome. Zeta potential values (mean ± SEM) are indicated for each formulation.


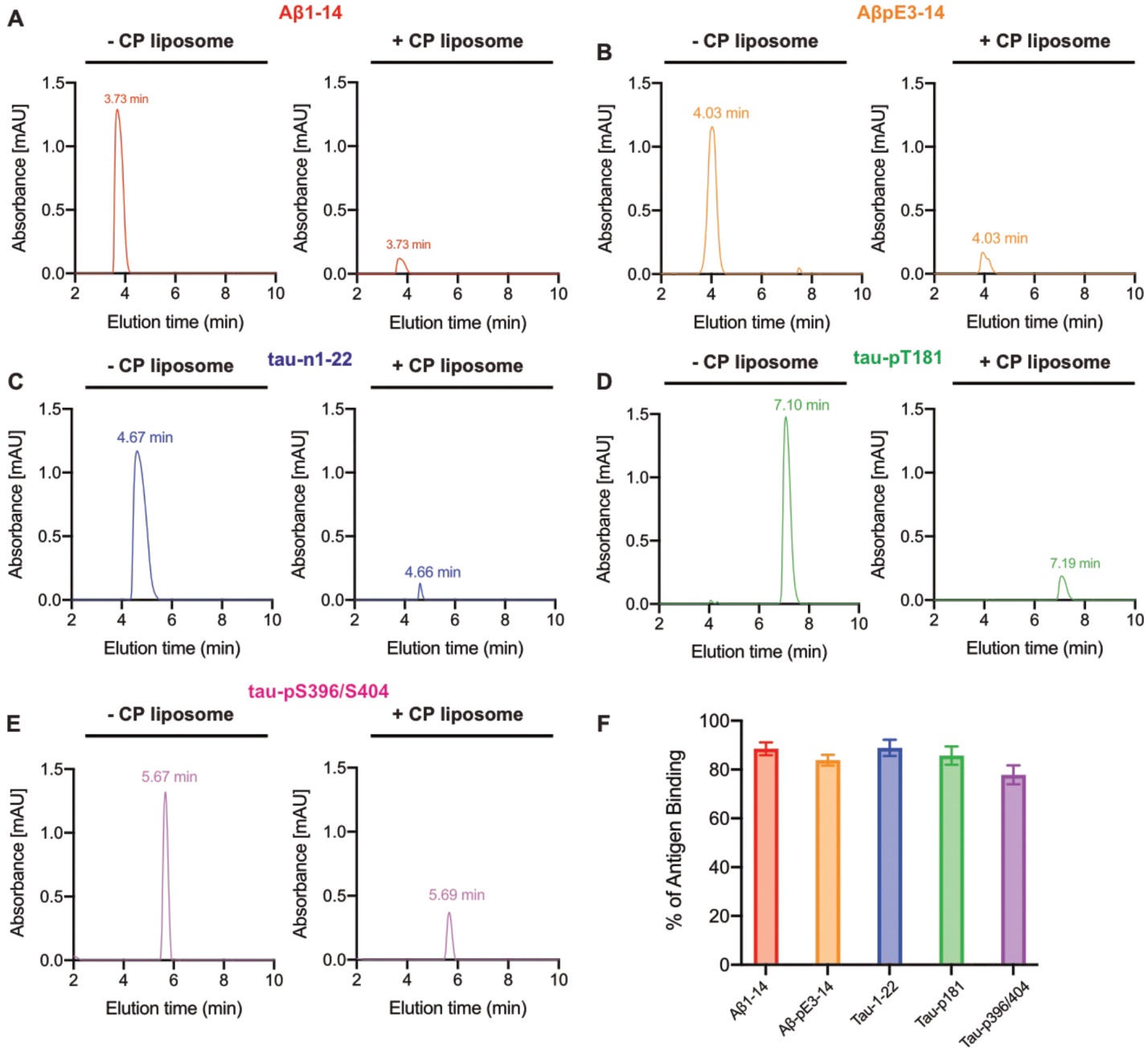


SI Figure 2. Representative Reversed Phase High Performance Liquid Chromatography (RP-HPLC) chromatographs of (**A**) Aβ1-14, **(B)** AβpE3-14, **(C)** tau-N1-22, **(D)** tau-pT181, **(E)** tau-pS396/S404 before (-) and after (+) bound to CP liposome with UV detection at 220 nm. **The analysis was made using C8 reverse phase column with 0.065% TFA in water and 0.05% TFA in ACN. (F)** Quantification of binding efficiency for each peptide to CP liposomes, expressed as % of antigen bound (mean ± SD, n = 3).


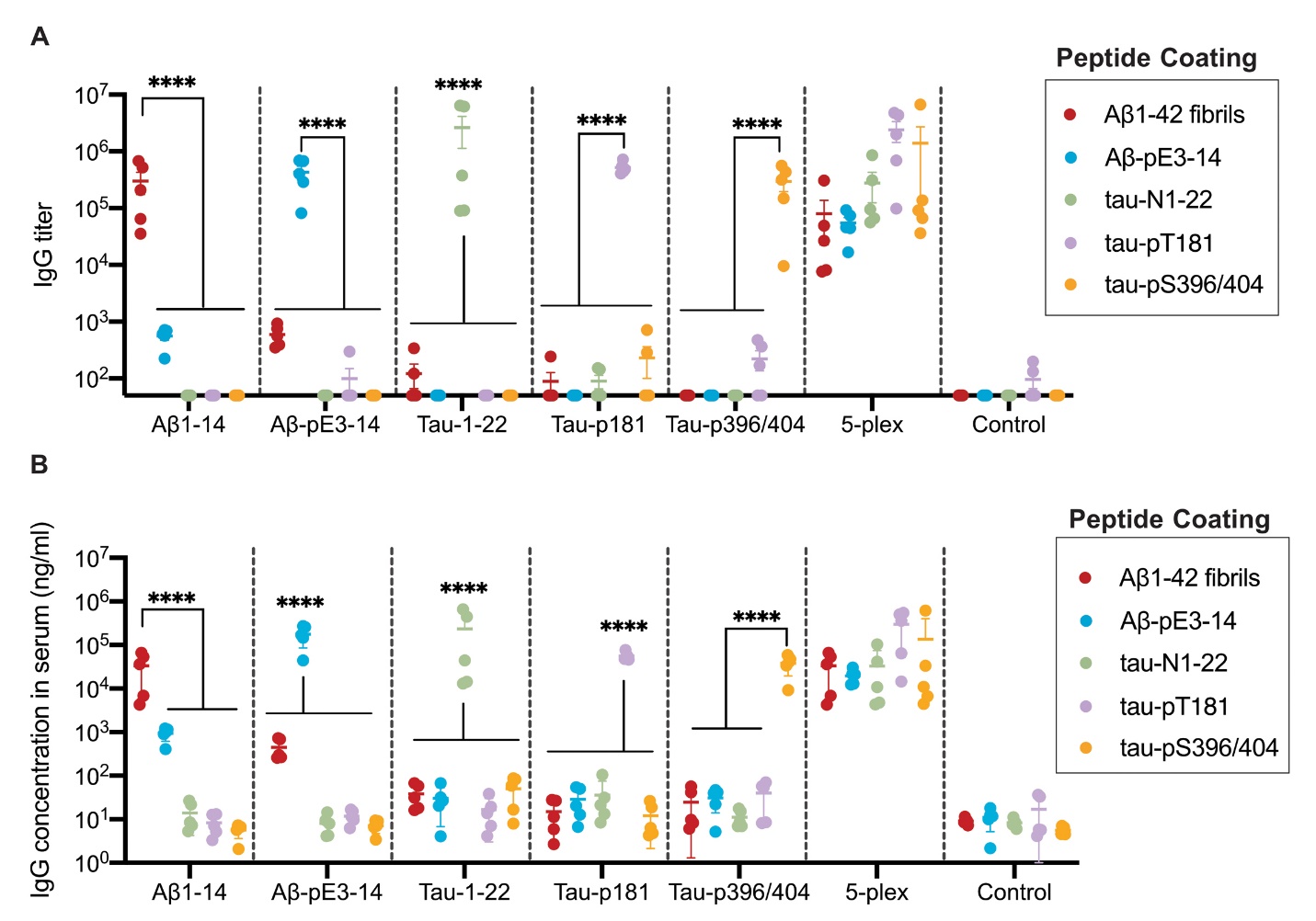


SI Figure 3. Antigen-specific IgG immune responses induced by SNAP-AD_5_ vaccination. **(**A**)** Serum from mice (n=5) were collected to assess IgG titers against individual antigen or 5-plex antigen mixtures by ELISA. **(**B**)** IgG concentration in mice serum (ng/mL) were quantified by ELISA using data from a commercial mAb IgG as standard curve. Error bars shown mean ± SD for n = 5, *****P<0.0001*, as determined by one-way ANOVA followed by Tukey’s test using log-transformed values.

**Supplementary table 1. Sample size for Figure 6D and E.**

| Cytokines/chemokines | Sample size | | | |
| --- | --- | --- | --- | --- |
|  | WT/Control | WT/SNAP-AD₅ | 3xTg-AD/Control | 3xTg-AD/SNAP-AD₅ |
| IL-1α | 13 | 16 | 17 | 17 |
| IL-1β | 9 | 5 | 11 | 10 |
| IL-2 | 14 | 14 | 14 | 14 |
| IL-5 | 10 | 12 | 15 | 15 |
| IL-6 | 10 | 9 | 12 | 14 |
| IL-12p70 | 10 | 11 | 12 | 14 |
| IL-17A | 11 | 14 | 15 | 14 |
| TNFα | 10 | 15 | 15 | 14 |
| IFNγ | 14 | 12 | 14 | 14 |
| MCP-1/CCL2 | 11 | 13 | 12 | 14 |
| KC/CXCL1 | 14 | 15 | 16 | 17 |
| MIP-2/CXCL2 | 14 | 16 | 16 | 16 |
| IL-4 | 8 | 13 | 11 | 14 |
| IL-10 | 10 | 12 | 13 | 15 |
| IL-13 | 12 | 16 | 13 | 15 |
